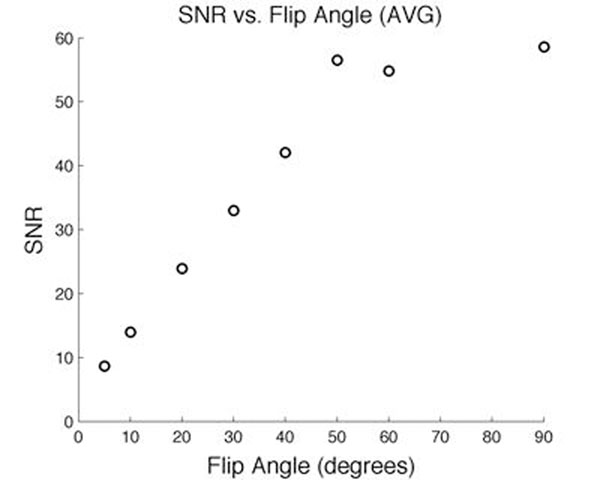# Flip angle optimization for quantitative phase contrast MR imaging

**DOI:** 10.1186/1532-429X-13-S1-P67

**Published:** 2011-02-02

**Authors:** Daniel B Ennis, Matthew J Middione

**Affiliations:** 1 University of California, Los Angeles, Los Angeles, CA, USA

## Objective

The overall objective of the work proposed herein is to optimize the flip angle in gradient-echo based PC-MRI methods for decreased measurement variability in quantitative blood velocity measurements of the common femoral arteries.

## Background

PC-MRI is a noninvasive imaging technique used to measure the velocity of flowing blood in a particular blood vessel with flexible spatial and temporal resolution [1,2]. It is considered the clinical “gold standard” for quantification of blood flow. PC-MRI boasts a variety of established applications in quantifying cardiovascular function and hemodynamics. In particular, this technique offers clinicians with a means of measuring peak velocity, mean velocity, flow rate, and total flow throughout the vasculature of the human body. One of the challenges in PC-MRI is the wide range of variables that make up a given protocol requiring careful optimization of each imaging parameter in an effort to accomplish the goals of a given scan. Surprisingly, the current PC-MRI utilizes a default flip angle, which has not been optimized. A theoretical description of the signal acquired from flowing spins in spoiled gradient-echo pulse sequences has been previously developed [3]. These efforts indicate that there exists a flip angle that provides an optimized SNR for a given tissue, echo time, repetition time, slice thickness and velocity, which leads to increased reproducibility of quantitative PC-MRI measurements. This optimization cannot be performed empirically because of the number of parameters involved, hence mathematical and computation work is required.

## Methods

All measurements were performed on a 1.5 Tesla system (Avanto; Siemens Medical Solutions, Erlangen, Germany) using a 16-channel surface coil. Blood velocity data in the common femoral arteries were obtained in 5 healthy consenting volunteers (4 male, 1 female, age __ ± __ years). PC-MRI was performed without breath-hold using retrospective ECG triggering. Imaging parameters included 340 x 212.5 mm field of view, 256 x 160 matrix, 1.3 mm isotropic resolution, 8 mm slice thickness, 5.4/3.2 msec TR/TE, 32.4 msec temporal resolution, a range of flip angles for optimization (5,10,15,20,30,40,60,75 and 90°), 888 Hz/pixel acquisition bandwidth, three views per segment and a velocity encoding strength of 75 cm/sec (53 second scan time). The imaging was repeated 5 times in each volunteer.

## Results

**Figure 1:**
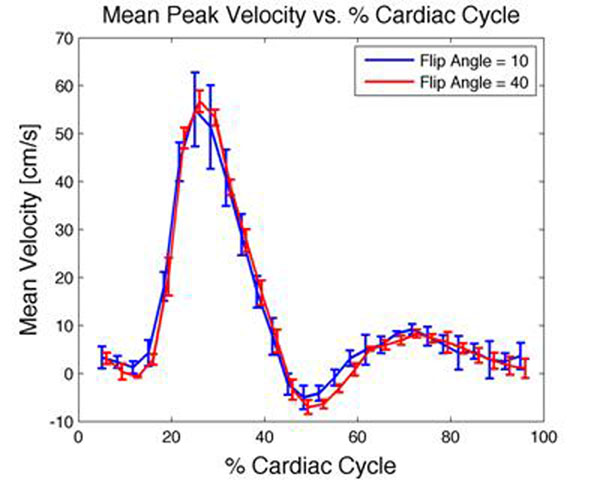


The results indicate that there exists an optimal flip angle (Figure 1) in which the SNR is optimized for a given experiment. The use of this flip angle yields decreased variability in quantitative blood flow measurements compared to other non-optimized flip angles (Figure 2). From these results it can be concluded that careful selection of the flip angle is required. Flip angles for flow quantification are not sequence dependent; rather they are velocity dependent for a given protocol and simply selecting a default flip angle for any given study will not be sufficient to yield the best results.

**Figure 2:**